# TIS Transformer: remapping the human proteome using deep learning

**DOI:** 10.1093/nargab/lqad021

**Published:** 2023-03-03

**Authors:** Jim Clauwaert, Zahra McVey, Ramneek Gupta, Gerben Menschaert

**Affiliations:** Department of Data Analysis and Mathematical Modelling, Ghent University, Ghent, Oost-Vlaanderen 9000, Belgium; Novo Nordisk Research Centre Oxford, Novo Nordisk Ltd., Crawley, South East England, RH6 0PA, UK; Novo Nordisk Research Centre Oxford, Novo Nordisk Ltd., Crawley, South East England, RH6 0PA, UK; Department of Data Analysis and Mathematical Modelling, Ghent University, Ghent, Oost-Vlaanderen 9000, Belgium

## Abstract

The correct mapping of the proteome is an important step towards advancing our understanding of biological systems and cellular mechanisms. Methods that provide better mappings can fuel important processes such as drug discovery and disease understanding. Currently, true determination of translation initiation sites is primarily achieved by *in vivo* experiments. Here, we propose TIS Transformer, a deep learning model for the determination of translation start sites solely utilizing the information embedded in the transcript nucleotide sequence. The method is built upon deep learning techniques first designed for natural language processing. We prove this approach to be best suited for learning the semantics of translation, outperforming previous approaches by a large margin. We demonstrate that limitations in the model performance are primarily due to the presence of low-quality annotations against which the model is evaluated against. Advantages of the method are its ability to detect key features of the translation process and multiple coding sequences on a transcript. These include micropeptides encoded by short Open Reading Frames, either alongside a canonical coding sequence or within long non-coding RNAs. To demonstrate the use of our methods, we applied TIS Transformer to remap the full human proteome.

## INTRODUCTION

Translation is the synthesis of proteins from messenger RNA (mRNA). The nucleotide sequence of mRNA not only encodes proteins through its codon structure, but also influences other factors, such as the location and efficiency of the translation process (1). The complexity of the nucleotide sequence is highlighted by the variety of gene products, playing roles in multiple molecular processes. Partly due to this complexity, existing mappings of the genome, transcriptome and proteome are still mostly based on the combination of experimental data with statistical tests. Genome annotation platforms such as Ensembl, NCBI and USCS rely largely on sequence alignment methods (2,3). Flaws of such systems are inherent to the knowledge at hand, where biases and errors are known to be propagated with time (4). Subsequently, changes in existing annotations are made as new data on gene products or molecular mechanisms become available.

A comprehensive understanding of translation in combination with the design of high performing machine learning approaches can advance the identification of novel proteins. In recent years, machine learning models have gained attention due to their ability to attain high performances when sufficient data is available. Prior work has attempted to predict TISs sites solely based on intronless transcript sequences (5–11). Deep learning techniques have also gained success due to their automated feature learning (12), as can be seen through the application of neural networks on sequence and omics data. Zhang *et al.* use a combination of convolutional and recurrent layers to process a transcript sequence of fixed length around the TIS (8). Similarly, Zuallaert *et al.* and Kalkatawi *et al.* have applied convolutional neural networks to determine the location of TISs (9,10). Although the predictive performance of these models still imposes restrictions on their wider applications, value from these studies is derived from insights gained on the underlying decision-making process.

Deep learning techniques have the unique strength of uncovering complex relationships in big data. Standard machine learning methods and traditional neural network architectures map relations of the input features to the target label. These relations are, for most architectures (e.g. linear regression, fully-connected layers, convolutions), explicitly mapped as trained weights that determine how information is combined in each layer. In contrast, attention methods determine how information within inputs is combined based on the inputs themselves and their (relative) positioning. This is calculated on the fly for each new set of inputs at each layer of the network. For many natural language processing tasks, this approach has proven superior (13–15). Similar to natural languages, information within biological sequences is complex and convoluted. Some recent studies have successfully shown the efficacy of transformer networks on genome data, such as proposed by Zaheer et al., for the prediction of promoter regions and chromatin profiling (16). Ji *et al.* introduce DNABERT, a transformer model pre-trained on genome data that can be fine-tuned for a specific task such as the annotation of promoters or splice sites (17).

The computational cost of attention imposes limitations on the maximum input length of sequential data. This cost scales quadratically with respect to the length over which attention is calculated. This has traditionally limited the maximum sequence length of previous studies to 512 units (15,17), making the application of recent transformer networks such as DNABERT not suitable for processing full transcript sequences. However, several recent studies have focused on overcoming this limitation through the use of mathematical approximation techniques that save on computational cost, allowing the attention mechanism to be applied over larger sequences (18–20).

**Figure 1.**
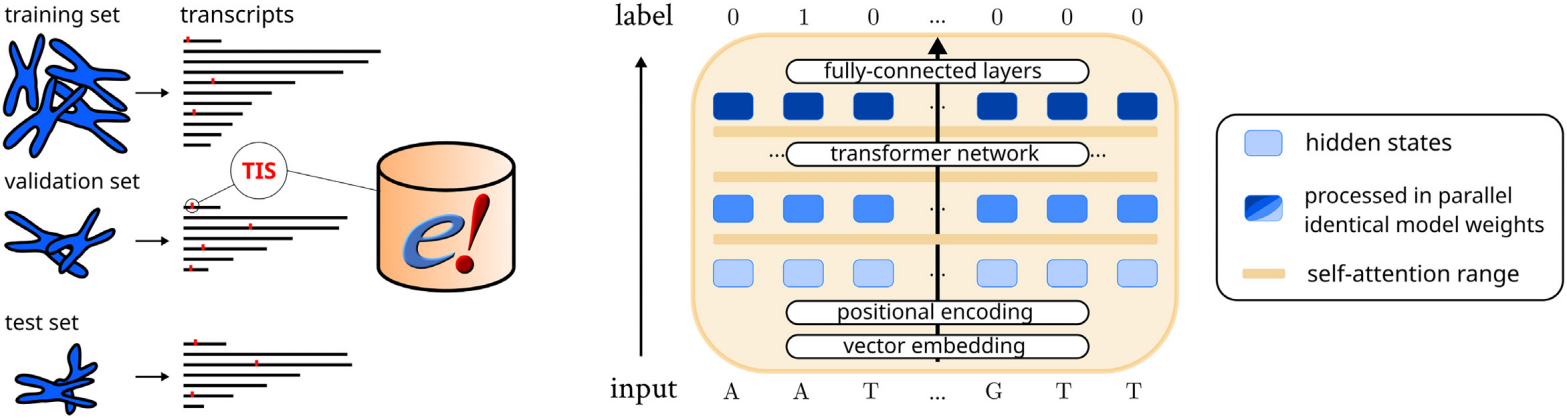
Schematic of the data and model set-up. (*Left*) The Ensembl annotation (version 107) is used to determine transcript sequences and translation initiation sites (TISs). Transcripts are grouped by chromosome to create a training, validation and test set. (*Right*) The performer model allows processing of full transcript sequences, evaluating data through the layers in parallel to obtain model outputs at each position. The model architecture can handle varying input lengths, as identical model weights are applied to transform the data. Through self-attention, sequential information from any site on the transcript can be queried by the model to determine the presence of TISs at any position.

In this work, we propose TIS Transformer (see Figure 1), a tool for the identification of TISs using the full processed transcript sequence as input. Our technique uses one of the proposed scaling solutions for computing attention (20), where transcripts up to a length of 30 000 nucleotides can be processed at single nucleotide resolution. Models are trained and evaluated on the processed human transcriptome, excluding introns and intergenic regions. First, we benchmark our method against previous studies, where we show state-of-the-art performances. Afterwards, we provide an in-depth analysis on the current state of applying learning methods for the annotation of TISs on the full human transcriptome. We show our approach to achieve notable performances in general, where learning and evaluation approaches are mostly hindered by low quality annotation. We believe this approach to be of substantial benefit to the community, where it can aid the discovery and annotation of novel proteins, thus building state-specific proteome maps, or the engineering of transcripts.

## MATERIALS AND METHODS

### Training objective

The goal of the study is to create a predictive model that is able to detect the occurrence of coding sequences (CDSs) within the transcriptome. Rather than predicting a paired TIS and translation termination site (TTS), we decided to achieve this goal through the detection of TISs, as it poses a simpler optimization and decision-making problem. TTSs have been determined post-hoc through detection of the first in-frame stop codon. As such, the model is optimized to perform binary classification between TISs and non-TISs for each nucleotide position of the human transcriptome. We provide all code material and results as part of the study.

### Model input and output

The model processes the full sequence of the transcript to impute the presence of TISs at each nucleotide position (see Figure 1). The input of the model is represented by $\boldsymbol {H}^{\text{in}} \in \mathbb {R}^{n \times d}$, where *n* < 30 000 and *d* the dimension of the hidden states. $\boldsymbol {H}_{i}^{in}$ denotes the nucleotide vector embedding (A, T, C ,G , N, [START], [STOP]) at position *i* of the transcript (thymine is used to proxy uracil throughout the paper). Vector embeddings are numerical representations (i.e., vectors) of concepts that exist within a multi-dimensional space, where their distance w.r.t. other embeddings (distance, angle) allow meaningful computations. The input embeddings are trained as part of the optimization process. The [START] and [STOP] tokens are used at before the beginning and after the end of the transcript, relatively. Identical in dimensions to the input embeddings, the output $\boldsymbol {H}^{\text{out}} \in \mathbb {R}^{n \times d}$ of the transformer network is processed by a pair of feed-forward layers which result in a model output at each nucleotide position of the transcript. The cross-entropy loss is computed from predictions at inputs ∈ {*A*, *T*, *C*, *G*, *N*}.

### Transformer architecture

The transformer architecture is a recent methodology that implements (self-)attention for a group of inputs. The attention mechanism is applied to determine the flow of information (i.e. applied weights to combine information) based on the information itself. The transformer network is characterized by several sequential layers with identical designs.

The principal element is the use of self-attention, performed by a module called the attention head. Here, a query, key and value matrix are derived from the input *X* in each layer:


(1) $$\begin{equation*} \boldsymbol {Q}, \boldsymbol {K}, \boldsymbol {V} = \boldsymbol {XW}_q^{\top }, \boldsymbol {XW}_k^{\top }, \boldsymbol {XW}_v^{\top }, \end{equation*}$$

where $\boldsymbol {Q}$, $\boldsymbol {K}$, $\boldsymbol {V} \in \mathbb {R}^{n \times d_k}$. For the first layer, $\boldsymbol {X}$ equals $\boldsymbol {H^{\text{in}}}$. Multiplication and subsequent normalization between $\boldsymbol {Q}$ and $\boldsymbol {K}^{\top }$ return a *n* × *n* matrix, whose values determine the flow of information to (e.g. $(\boldsymbol {QK^\top })_{i,:}$) and from (e.g. $(\boldsymbol {QK^\top })_{:,i}$) each hidden state through multiplication with $\boldsymbol {V}$. For each attention head, a matrix $\boldsymbol {Z}$ is calculated, holding the combined information for each input hidden state.


(2) $$\begin{equation*} \boldsymbol {Z} = \text{softmax}\left( \frac{\boldsymbol {QK}^{\top }}{\sqrt{d_k}} \right) \boldsymbol {V} \end{equation*}$$

where $\boldsymbol {Z} \in \mathbb {R}^{n \times d_k}$. Multiple sets of $\boldsymbol {W}_q, \boldsymbol {W}_k, \boldsymbol {W}_v$ make it possible to route multiple types of information from $\boldsymbol {X}$ in each layer. The outputs $\boldsymbol {Z}$ that are derived from different attention heads are concatenated and transformed to fit the dimension size of the input $\boldsymbol {X}$, with which they are summed (i.e. residual connection). Each layer is built using the same components, albeit unique weights. The architecture allows processing of transcripts of different lengths, as the same weights are applied to calculate the $\boldsymbol {q}$, $\boldsymbol {k}$ and $\boldsymbol {v}$ vectors for each input hidden state (independent of position). These are calculated in parallel for each layer and represented as rows in the matrices $\boldsymbol {Q}$, $\boldsymbol {K}$ and $\boldsymbol {V}$. Positional encodings are used to incorporate positional information with each input. These vectors are of the same size as the inputs with which they are summed and can be utilized as biases that are fixed for each position. Previous research has shown that the model can utilize this information (15).

To allow long-range attention over full transcripts, we used a recent innovation in calculating full attention introduced by Choromanski et al. (20). Equation (2) is exchanged with the Fast Attention Via Positive Orthogonal Random Features (FAVOR+) algorithm, which utilizes random feature maps decompositions for approximation in order to obtain a computational complexity that scales linearly with respect to the length of the sequence. The final model uses a combination of local and full attention heads (Supplementary Table A6, Supplementary Figure A14). In contrast to full attention, local attention limits the input range over which attention is calculated to a smaller window.

### Data sets and model optimization

No single data set has previously been designed to function as a benchmark standard when evaluating the prediction of TISs. The Ensembl assembly of the Human genome (GRCh38.p13; release 107) was selected to provide the transcript sequences and label the known TISs. The data set contains a total of 431 011 438 RNA nucleotide positions, 96 655 of which are positively labeled as a TIS (0.022%), indicating an extremely imbalanced class distribution. The model processes full transcripts in parallel, of which 96 655 (38.49%) are protein-coding and 154 ,466 (61.51%) are non-coding. The training, validation, and test set are allocated in accordance with chromosomes, thereby grouping transcript isoforms and proteoforms together. Transcripts longer than 30 000 nucleotides (17 instances) are excluded due to memory requirements. When remapping the full human proteome, six models were trained to sequentially use different sets of chromosomes as test, validation, and train data (Supplementary Table A2 and Figure 1). The annotations on the test sets, constituting one sixth of the chromosomes for each model, are used to obtain a complete re-annotation. To perform the benchmark, several additional constraints were required to ensure all methods were trained and evaluated utilizing the same data (see Supplementary Files). To illustrate, these exclude non-ATG positions and positions at the edge of transcripts that cannot be parsed by one or more of the evaluated methods. The benchmark was performed using chromosome 1, 7, 13, 19 as testing data and chromosome 2 and 14 as validation data. This results in train, validation and test sets of 3 608 307, 641 264 and 1 069 321 candidate TIS positions, respectively. For all instances, training was stopped when a minimum cross-entropy loss on the validation set is obtained. All reported performance metrics are obtained on the test sets. Before benchmarking our method with other studies, hyperparameter tuning was performed (Supplementary Figure A4). In general, it was observed that the performance of the model was not correlated substantially with any single parameters, but with the total number of model parameters. The number of model parameters is influenced by the dimension of the hidden states ($\boldsymbol {H}$), number of layers, dimension of the attention vectors *q*, *kandv* and number of attention heads. Through hyperparameter tuning, two model architectures were selected to benchmark against previous approaches, TIS Transformer and TIS Transformer L(arge), each featuring 118K and 356K model parameters. Supplementary Tables A3– A5 list the details of each model architecture.

## RESULTS

The study is separated into two main parts. First, we perform a benchmark to compare our method with previous studies and find our method to achieve state-the-art performances. Afterwards, we remapped the human proteome using our approach for a more in-depth analysis on the ability of our models.

### Benchmark

A multitude of studies exist that apply machine learning techniques for the prediction of TISs (Supplementary Table A1) (5–11,21,22). Two main approaches exist that model sequence information to the occurrence of TISs: support vector machines and neural networks. Problematically, the computational cost for support vector machines scales quadratic with the number of samples evaluated. As such, given tools are impossible to be applied on the full genome. To illustrate, the maximum data set size applied by previous studies is 13 503 samples, <0.01% of the positions on the full transcriptome. The limited data size allowed to train these models is an important disadvantage, where previous studies show that support vector machine implementations are consistently outperformed by neural network approaches (9,10,21).

Candidate TIS positions are identical for the training and evaluation of different approaches to ensure a fair comparison. The construction of the training, validation and test set is discussed in more detail in Section 2.2 of the Supplementary Files. We rely on previous results that show listed neural networks approaches to outperform support vector machines, as proposed data set sizes cannot be applied to train support vector machines. Furthermore, we were unable to incorporate the method DeepTIS, as proposed by (21), due to missing details on the model architecture in the published paper. See the Supplementary Files for more information on, and an extended discussion of, the individual methods and the benchmark.

Results show TIS Transformer to largely outperform previous methods (Table 1, Supplementary Figure A13). As TIS Transformer L(arge) performs better on the benchmark data set, it is the model architecture that has been applied to remap the human proteome.

**Table 1.** Benchmarked performances on a set of available tools. For each study are given: the year of publication, the nucleotide input size parsed by the machine learning model, the total number of trainable parameters, the training time until convergence, and the ROC and PR AUC scores. Note that methods applying support vector machines could not be evaluated due to the size of the data, nor DeepTIS due to missing information. The total number of transcript positions used to train, validate and test each method count to a total of 3 608 307, 641 264 and 1 069 321 positions

Model name	Year	Input size	Parameters	Train time	ROC AUC	PR AUC
DeepGSR (10)	2019	600nt	∼181M	∼100h	0.9485	0.2110
TITER (8)	2017	203nt	∼431K	∼24h	0.9617	0.3770
TISRover (9)	2018	203nt	∼240K	∼3h45	0.9650	0.4495
TIS Transformer	2022	Transcript	∼118K	∼10h	0.9970	0.7628
TIS Transformer L	2022	Transcript	∼356K	∼6h	0.9971	0.7684

**DeepTIS** (21): see supplementary files on missing details on its architecture.

**SVM** (5–7,11): training of support vector machines is not tractable on large data sets.

### Evaluation of the remapped proteome

#### Performance evaluation

**Figure 2.**
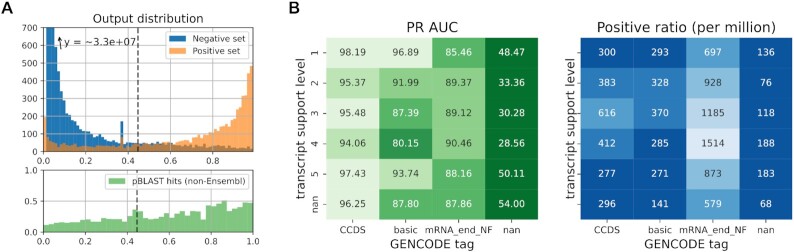
Model performances on predicting translation initiation sites. (**A**) (top) A histogram of the model output predictions when evaluated on chromosome 2 (test set). Transcript sites are divided in a negative (blue) and positive (orange) set according to the annotations provided by Ensembl. The dotted line represents the threshold that an equal number of positive model predictions as provided by Ensembl. (bottom) The resulting coding sequences of predicted TISs were evaluated against UniProt using pBLAST. Shown are the fraction of coding sequences returning a good match in relation to the model output score. Only TISs not annotated by Ensembl (i.e., negative set) were considered. (**B**) Model performances were binned according to transcripts properties. PR AUC performances (left) and the ratio of positive samples in each group (right) are obtained by binning the transcripts according to transcript support level and other properties given to the annotated translation initiation site or transcript (if any, otherwise nan).

To perform an in-depth analysis on the model predictions of the full human transcriptome, a cross-validation scheme is used (see Section 2.4, Supplementary Table A2, Supplementary Figures A5 and A6). The average area under the precision-recall curve (PR AUC) score of the six models trained to remap the proteome is 0.839. These scores underline the capability of the TIS Transformer to predict with a high recall and precision given the extremely imbalanced setting, in turn reflecting the usefulness of the model to be applied to predict sites of interest on the full transcriptome. The output probabilities of the negative and positive samples can be clearly distinguished as they are concentrated at 0 and 1, respectively (Figure 2A). Similarly, this can be observed when evaluating the model outputs at the single transcript level (e.g. see Figure 4).

**Figure 3.**
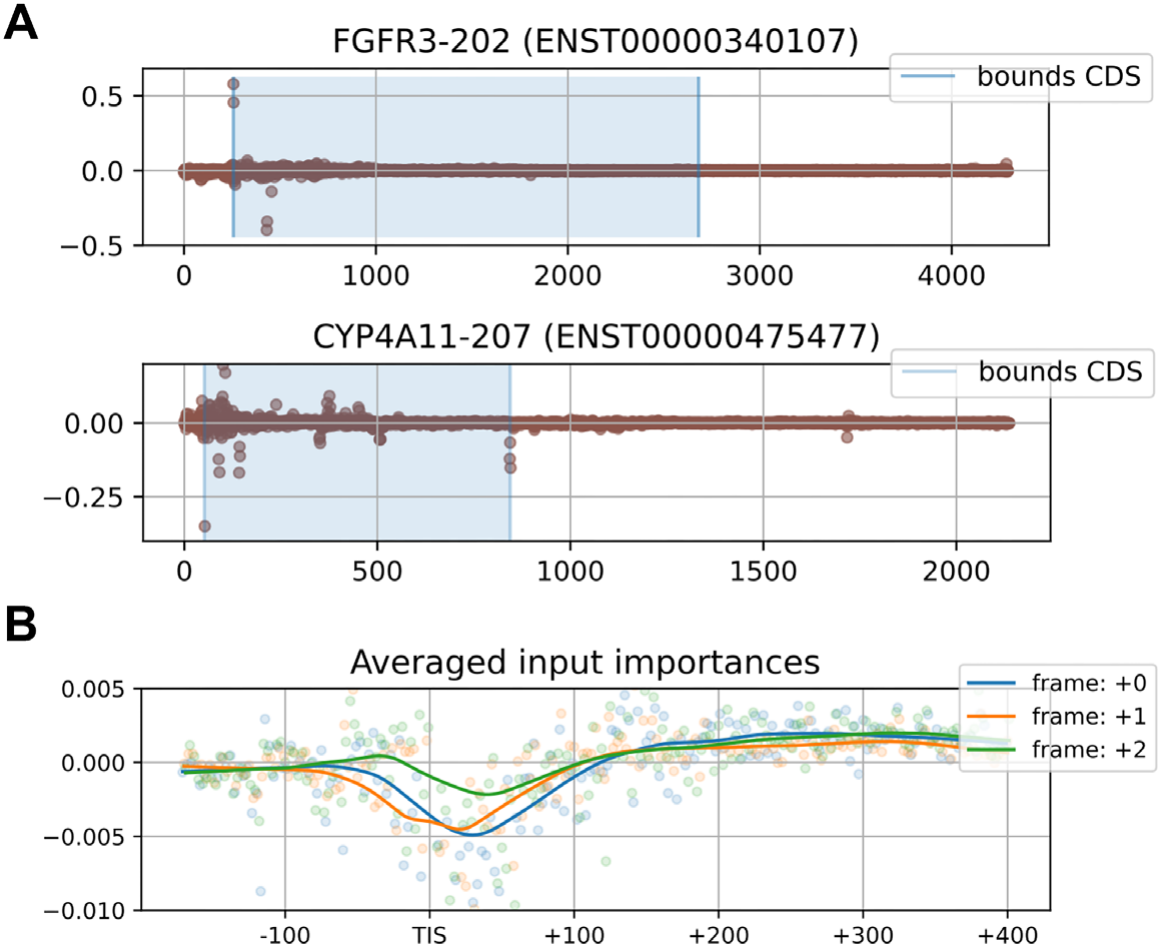
Attribution scores of input nucleotides which reflect the importance of each nucleotide towards predicting the annotated TIS. (**A**) Scores shown are given by the model for the translation initiation site on position 257 of FGFR3 (ENST00000340107) and at position 52 of CYP4A11 (ENST00000475477). (**B**) Attribution scores for the positions surrounding the translation initiation site (TIS) averaged for all top ranked predictions. a rolling average is given for the three reading frames.

**Figure 4.**
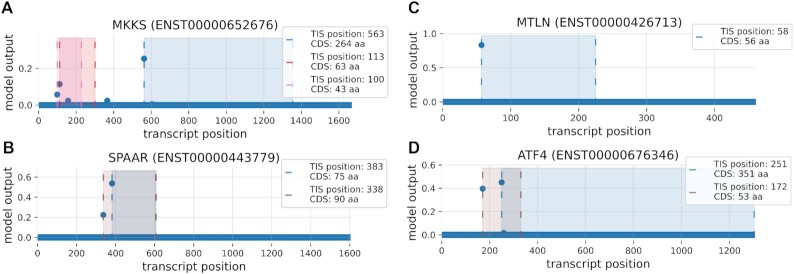
Example transcripts with predicted micropeptide expression. Shown are the model outputs (y-axis) for each position of the transcript (x-axis). For high TIS predictions, the bounds (striped line) and area of the resulting CDSs are shown. Given are the predictions for the transcripts. (**A**) MKKS (ENST00000652676), (**B**) SPAAR (ENST00000443779), (**C**) MTLN (ENST00000426713) and (**D**) ATF4 (ENST00000676346).

Several patterns exist between the model performance and data properties as obtained by GENCODE (Figure 2B). Some properties are linked to the transcript, such as the transcript support level, which quantifies the support for a transcript to exist based on available *in vivo* evidence (23). Other discussed properties relate to whether these TISs are part of the consensus coding sequence database (CCDS), whether the transcript and resulting coding sequence is identified as a representative sample for the gene (basic), and whether the mRNA end could be confirmed or not (mRNA_end_NF). Supplementary Figure A7 includes more information for each group including the ROC AUC scores and number of nucleotide positions (samples) in each group.

We observe several patterns of special interest. First, the performance of the model is correlated to the transcript support level for the bulk of annotations (CCDS, basic). Only the group with the lowest transcript support level (i.e. 5) shows better performances than expected based on its ranking. As this group of transcripts is described as ‘no single transcript supports the model structure’, we hypothesise their presence to be supported by other factors such as computational pipelines, which can be expected to have good performance. Second, the model performs worse for the group of transcripts where no tags are given to the transcript or TISs. These tags include the validation of TISs by other parties such as the CCDS initiative. Third, the differences in performance between groups is largely uncorrelated to the ratio of positive samples along rows and columns, which could otherwise have been the driving factor for differences in PR AUC scores.

Figure 2A shows the relation between the model output and the percentage of resulting CDSs having been observed by previous studies but are not included in Ensembl. These show a clear correlation between the model output and the percentage of positive matches. These results were obtained by querying CDSs resulting from high-ranking predictions that were not previously annotated by Ensembl against Swiss-Prot and TrEMBL (mammals) using pBLAST. These highly predicted positions are present on both transcripts that currently have annotated TISs and those that are labaled as non-coding transcripts. Supplementary Figure A12 includes several transcripts with high ranking predictions that currently do not contain annotated TISs. Section 3.2 of the Supplementary Files lists the required conditions for a match to be considered valid. Corroborating previous results, the fraction of pBLAST matches is also correlated with the quality of the transcript support level (Supplementary Figures A8 and A9). Supplementary Figure A12

#### Input attribution score analysis

Several techniques exist that allow us to gain insights into the decision-making process of the trained model. Here, we apply integrated gradients to evaluate the relative contribution of the input nucleotides on the transcript to the predicted output (Figure 3A). Integrated gradients, first introduced by Sundararajan et al., utilizes the partial derivatives of the model prediction with respect to the input values in order to assign attribution scores (24). We observe several expected patterns, such as the high importance of the candidate TIS itself and surrounding areas (e.g. Kozak sequence context). In addition to this, we see recurring patterns that both the translation termination sites and reading frame can have importance towards determining TISs (Figure 3B). We hypothesize that the relatively higher importance of the second and third nucleotide within each codon of the reading frame can be ascribed to their relatively higher information content (in relation to the defined amino acid) as compared to the first nucleotide of each codon. No annotations on reading frames or translation termination sites were given to the model, reflecting the capability of the model to determine complex features. It is possible that the importance of the codon content and translation stop sites is linked to the model learning general properties of relevant coding sequences on top of biological factors that determine translation to start. These might be relevant to detect special-type translation initiation sites, such as those with short or missing 5’ untranslated regions, but might also be a result of incorporating existing biases (e.g. CDS length).

#### Detection of small proteins and multi-TIS transcripts

In 1994, it was postulated that the minimum length of functional proteins is around 100 amino acids (25). Today, multiple studies have reported proteins shorter than 100 amino acids (26,27), fulfilling roles in different types of regulatory mechanisms (28,29). Nonetheless, small open reading frame encoded peptides (SEPs) translated from small open reading frames (sORFs) continue to be underrepresented in existing annotations (30). With sequence alignment algorithms suffering from low statistical power for shorter sequences, more evidence is needed to differentiate the false from the true positives (31).

The introduction of high-performing machine learning models could offer a solution to the detection of SEPs. The ability of the model to detect SEPs is reflected by the overlap between model predictions and a recently published list of newly introduced SEPs that were discovered using ribosome profiling (32) (see Supplementary Table A6). Nonetheless, the absence of SEPs in the data used to train and validate novel approaches is likely to influence the model and evaluation process. We observe overall lower probability scores for TIS positions that result in smaller CDSs (Supplementary Figure A11).

Figure 4 shows some examples on the output of the TIS Transformer for several high-profile and validated SEPs. Akimoto *et al.* prove the existence of three upstream open reading frames (uORFs) for the MKKS gene through proteome analysis, serving as a regulatory mechanism (peptoswitch) (33). uMKKS0, uMKKS1 and uMKKS2 are reported to be 43, 63 and 50 amino acids long, respectively (Figure 4 A). Matsumoto et al. could validate the expression of a 90 amino acid sORF on the SPAAR gene (Figure 4B) (34). The micropeptide is shown to be an important factor in regulating biological pathways related to muscle regeneration. Two studies reported the existence of a 56 SEP, found to affect mitochondrial respiration (35,36) (Figure 4 C). Young *et al.* report the existence of an upstream CDS of 53 codons overlapping with the ATF-4 coding region (Figure 4D) (37) . Supplementary Table A6 features more information on model predictions of recently recovered small ORFs (38). Supplementary Figure A12 gives the model output of transcripts with multiple high-ranking CDSs, some of which being short CDSs.

In contrast to the Ensembl database used for training, the model allows for multiple high ranking TIS per transcript (Figure 4A, B, D) and does not seem to be affected by the lack of multiple TISs in the training data. When selecting as many TISs within the positive set as are present in the Ensembl annotations (i.e. by incorporating the top-ranking predictions in the positive set), a total of 956 transcripts with multiple TISs would be obtained. Supplementary Figure A10 shows the occurrence of upstream (overlapping), downstream (overlapping), and internal ORFs on the transcriptome annotated by TIS Transformer. Several examples of such transcripts are shown in Supplementary Figure A12.

## DISCUSSION

Recent advancements of machine learning in processing sequential data, mainly introduced in the field of natural language processing, portend new opportunities towards the creation of predictive models on biological sequence data. The introduction of the FAVOR+ mechanism, which reduces the complexity of the attention step, has been an essential advancement to make processing of full transcript sequences at single nucleotide resolution possible. In this study, we investigate the use of these transformer networks to determine TISs based on the processed transcript sequences. We introduce TIS Transformer and benchmark it with previous solutions and show that a major improvement in performance was achieved. Transformers offer several advantages, notably, the model is able to parse variable length inputs, allowing it to process the full transcript sequence. It is efficient in parsing transcript information, as it requires only to parse the full transcript once to provide predictions for all its nucleotide positions. Furthermore, we see that improved performances have been achieved with a similar number of model weights as compared to previous tools. Although the research objective has been taken on by a plethora of studies in the past, the general usability and advantage of utilizing machine learning for annotating the full transcriptome has remained unclear. Various factors that previously posed as limitations, such as computational requirements, do not necessarily prove to be an inhibiting factor today.

### Ensembl annotations

Selection of the Ensembl data set to perform the study was based on multiple arguments. It currently includes annotations from several other collaborations such as CCDS and GENCODE. Featured annotations are not derived from a single experiment or generated using a single algorithm. Rather, the database is the result of multiple decades of careful curation and is based on the work of thousands of independent experiments. Unfortunately, it is plausible that our current knowledge of the human proteome is biased and incomplete. A lack of annotated SEPs and the existence of multi-TIS transcripts have previously been hypothesized (30,33). TISs that result in products that are hard to detect using common experimental methods (e.g., too small, unstable/aberrant proteins) are bound to be underrepresented in today’s data sets. These biases cannot be simply solved as part of this research, but will require an iterative and targeted effort.

We could determine no biological factors that could explain the correlation between the performance of the model and the transcript support level (Figure 2B; Supplementary Figures A8 and A9) or GENCODE tag (Figure 2B). In contrast, the ability of the model to detect novel TISs is corroborated by the increasing fraction of validated TISs using pBLAST (Figure 2A). Overall, these findings give a strong indication that performances are at least partially caused by (i) data set limitations, such as the lack of multi-TIS transcripts, (ii) faulty transcript sequences resulting from errors in the transcriptome and (iii) noisy TISs annotations due to biases in the data set (e.g. SEPs).

### Transcript types

In this study, the positive set incorporates all TIS annotations provided by Ensembl, thereby also including the nucleotide positions on a set of transcript types that are actively regulated against or result in faulty protein products. Nonsense mediated decay is a mechanism that targets faulty mRNA transcripts and breaks these down to prevent the initiation of aberrant translation products (39). Another set of transcript isoforms misses validated coding mRNA ends (GENCODE tags CDS_end_NF/mRNA_end_NF) or retains introns. We see that a large portion of novel annotations performed by the model occurs on special type transcripts (see Supplementary Figure A10), following the idea that unstable proteins are underrepresented in today’s known proteome. Future work might focus in on differentiating between types of translation events, such as those resulting in unstable proteins, in order to evaluate the influence on the resulting model.

### Non-canonical start codons

The presence of non-canonical start codons has been shown by various studies through the analysis of ribosome profiling data (40), where near-cognate start codons make up a substantial part of the predicted TISs. Due to these findings, it is hypothesized that today’s TIS annotations are lacking for near-cognate start codons. The model conforms to the importance of ATG as a start codon, where only seven (0.006%) instances (all CTG) of non-cognate start codons are present within the top scoring predictions, including as many predicted TISs per chromosome as annotated by Ensembl. This behavior is likely explained by the lack of non-ATG start codons in the Ensembl annotations today.

### Future prospects

The relevance of deep learning has strongly increased in recent years as its application and adaptation becomes more widespread. Most notably has been the release of AlphaFold, which has become a central tool for protein structure prediction (41). Similarly, annotation software driven by machine learning, such as TIS transformer, can drive the design of future studies or serve as an extra validation step. These can be focused on the discovery of new proteins, improving our understanding of biological drivers of translation, or predicting the occurrence of TISs on novel transcripts, a required step in the study of biological pathways.

## DATA AVAILABILITY

The main repository is available at https://github.com/jdcla/TIS_transformer, https://doi.org/10.5281/zenodo.7611886. All annotations of the model are publicly available. All code is available and can be used to train varying model architectures or remap the proteome of other organisms. All discussed models are made available for the community and can thereby be applied to custom transcripts. The input data, unprocessed model outputs, and curated predictions for each chromosome, as used in this study, are available. Lastly, to promote access to the results, and allow users to quickly obtain predicted TISs given certain criteria, we provide an online tool that is linked on the GitHub and currently online at https://jdcla.ugent.be/TIS_transformer (Supplementary Figures A1– 3). To illustrate, one can easily collect the small ORFs on non-coding sequences, all transcripts featuring multiple TISs, and transcripts featuring upstream ORFs.

## Supplementary Material

lqad021_Supplemental_File
