# Supplementary material for: TIS Transformer: remapping the human proteome using deep learning
Source: NAR Genom Bioinform. 2023 Mar 3;5(1):lqad021. doi: 10.1093/nargab/lqad021 (PMC9985340; doi:10.1093/nargab/lqad021)
Supplement: lqad021_Supplemental_File [file lqad021_supplemental_file.pdf]

Supplementary Information and Figures

TIS transformer: Remapping the human proteome using deep learning

Jim Clauwaert [1a], Zahra McVey [2], Ramneek Gupta [2], Gerben Menschaert [1b]

[1] *BioBix, Department of Data Analysis and Mathematical Modelling, Ghent University, Coupure Links 653, 9000 Gent, Belgium and* [2] *Novo Nordisk Research Centre Oxford, Novo Nordisk Ltd., Oxford, United Kingdom*

1. Model

1.1. Model architecture

The model architecture consists of multiple layers that are identical in structure but feature unique trainable model parameters. Two sets of embeddings are used; trainable nucleotide embeddings (A, C, G, T, N, [START], [STOP], [MASK]) and fixed positional embeddings. The transformer structure features multiple layers with multiple attention heads per layer. The workings of the Performer network are described by Choromanski et al. [2021]. The outputs of the network are send to a set of fully connected layers to obtain a binary output at each input position.

Algorithm 1 TIS Transformer network architecture. Given are the different layers, their respective dimensions as defined by their hyperparameter names, the dimensions for TIS Transformer (Table A4), and the resulting total weights.

TIS Transformer | **118,070**
- **Nucleotide Embedding** | input tokens $\times$ dim | 8×30 | **240**
- **Positional Embedding** | fixed positional embeddings | **0**
- **Performer** | **115,848**
  - **Layer** ($\times$ depth | 6) | **19,308**
    - **Layer norm** | dim $\times$ 2 | 30×2 | **60**
    - **Attention head** ($\times$ n_head | 6) | **1,488**
      - $\mathbf{W_Q}$ | dim $\times$ dim_head | 30×16 | **496**
      - $\mathbf{W_K}$ | dim $\times$ dim_head | 30×16 | **496**
      - $\mathbf{W_V}$ | dim $\times$ dim_head | 30×16 | **496**
    - $\mathbf{W_o}$ | dim_head $*$ n_head $\times$ dim | 96×30 | **2,910**
    - **Layer norm** | dim $\times$ 2 | 30×2 | **60**
    - **Linear** | dim $\times$ dim $*$ 4 | 30×120 | **3,720**
    - **Linear** | dim $*$ 4 $\times$ dim | 120×30 | **3,630**
- **Linear** | dim $\times$ dim $*$ 2 | 30×60 | **1,860**
- **Linear** | dim $\times$ 2 | 60×2 | **122**

1.2. Attention

Custom attention strategies can be performed by the attention heads independent of the number of weights utilized to calculate the **Q**, **K**, **V** matrices. In this model, full attention is calculated through the Fast Attention Via Positive Orthogonal Random Features (FAVOR+) algorithm (Figure A13: left). These allow every input token to attend to all other inputs along the transcript. In contrast, local attention restricts the attention matrix to only neighboring positions. Local attention is implemented by dividing the attention matrix in smaller blocks on which full attention is calculated (Figure A13: right). Three blocks around the evaluated input are calculated. These local attention heads do not apply the FAVOR+ algorithm and use rotary positional embeddings Su et al. [2022]. The block size of the local attention heads is referred to as 'local_window_size' in Supplementary Table A4-A6.

2. Model selection

2.1. Hyperparameter optimization

Hyperparameter optimization is performed for a single set-up: transcripts from chromosomes 1, 7, 13, and 19 are excluded (test set) and chromosomes 2 and 14 are applied to select the optimal hyperparameters (validation set). Overall, no individual hyperparameters were observed to be more effective than others in improving performances. It was observed that a correlation exists between the total number of model parameters and model performance. To reflect this, the performances of three model architectures are given; TIS Transformer S(mall), TIS Transformer and TIS Transformer (L)arge (see Table A2, A3, A4, A5). Each network represents a tripling of model parameters. Performance gains showed to be most substantial when increasing the model parameters (i.e. number of layers, number and dimensions of attention heads,

[a]Jim Clauwaert. Tel.: +32 926 49922; Email: jim.clauwaert@ugent.be
[b]Gerben Menschaert. Tel.: +32 926 49922; Email: gerben.menschaert@ugent.be

dimension of the hidden state) up to a certain point, after which gains stagnate. Although three times bigger, the performance of the TIS Transformer L is marginally better than that of TIS Transformer. The minimal loss on the validation set for both architectures is similar before they both start overfitting (see Figure A4). These findings reflect those given in the main manuscript, where further improvements of machine learning approaches are likely to be hampered by a set of noisy annotations. This was shown through the correlation of performances with the support level of the transcripts and the verification of other annotation platforms such as CCDS.

Notwithstanding the size of the data set and overall high computational requirements of transformer architectures, model optimization is possible on a single RTX 3090 and converged after ca. 10 hours due to the relative shallowness of the final transformer architecture (Supplementary Tables A3–A5). The details of varying model architecture performances are given by Supplementary Table A2 and Supplementary Figure A4, A5, A6.

2.2. Benchmarking

A multitude of studies have previously been performed applying machine learning techniques for the prediction of TISs. Previous studies utilizing the transcript nucleotide sequence have been listed in Supplementary Table A1 Zien et al. [2000], Saeys et al. [2007], Chen et al. [2014], Kabir et al. [2015], Goel et al. [2020], Zhang et al. [2017], Zuallaert et al. [2018], Kalkatawi et al. [2019], Wei et al. [2021]. In contrast to existence of multiple studies, no single data set exists that functions as the go-to benchmark data set for this problem setting. This can be attributed to various reasons, the main ones being the computational limitations of some techniques making it impossible to process multiple millions of samples, and the obscurity of the ground truth, resulting in multiple existing platforms each featuring varying sets of annotated TISs.

In this study, we utilize the full genome of the selected organism (Homo sapiens) of Ensembl to train, validate, and test the model with. We believe this approach to have several advantages over custom sub-sampled data sets. The vast majority of transcript positions are non-TIS sites, where sub-sampling mainly affects the negative set. The technique used to sub-sample the negative set influences the population sampled, and thus the resulting performances. To illustrate, a model sampling ca. 10,000 samples of the negative set at random effectively covers only 0.002% of the population. For a setting where 0.01% of the negative samples bear sequence similarity to the region of an actual TIS position (i.e. hard to predict), this would result in five such samples in the negative set. Performances measured on such models will easily result in near-perfect precision and accuracy scores. However, it fails to portray the model's capability when applied on the full transcriptome, where the vast number of negative samples results in a set of false positives that heavily outweighs the number of positive samples. Most studies aim to balance the number of positive and negative samples. While several studies discuss an approach that seeks to sample the negative samples that have similarity with the positive samples (i.e. hard to predict), each study follows a new approach. As such, variations between various methods of sampling causes resulting model performances to vary. This is illustrated by our results contradicting published results. TITER performs better than DeepGSR as published by [Kalkatawi et al., 2019].

It is impossible to perform a benchmark against approaches utilizing support vector machines due to the size of the data. Large data sets are required to train for neural networks, but pose a problem for support vector machines, where the training and evaluation time scale quadratically with the number of samples processed. It is nonetheless implausible that support vector machines can offer comparable performances considering the low number of samples they are trained with. Additionally, all previous studies incorporating support vector machines as part of their benchmark list these models as inferior to neural network implementations [Zuallaert et al., 2018, Wei et al., 2021, Kalkatawi et al., 2019]. Lastly, we were unable to apply DeepTIS [Wei et al., 2021] due to a lack of information given in the paper. Some of the missing or unclear details include specific hyperparameter values (e.g. q which defines the input window length) or the exact model architecture of 'DeepTIS2', featured in the paper as the best performing one. The online GitHub repository seemed incomplete as there was no code utilizing recurrent neural networks, which should be part of the backbone of 'DeepTIS2'. We were unable to reach the authors of this work for clarification.

To ensure a fair comparison of the listed methods, all methods are being trained and evaluated on the exact same data sets. Due to several constraints imposed by individual methods, all transcript positions matching at least one of these constraints are excluded from the data. These constraints are: only ATG sites, only positions on transcripts with a length of less than 30,000 nucleotides, only transcript positions that are distanced at least 300 nucleotides from the start and end of the transcript, and no 'N' annotated nucleotides within a 300nt window of the candidate TIS site. Applying these constraints on the full results in a train, validation and test set of 3,608,307, 641,264 and 1,069,321 candidate TIS positions. Due to several available network architectures being implemented using outdated software packages for GPU accelerated computation (TISRover: Lasagne, TITER: Theano), the decision was made to re-implement all models using PyTorch (Lightning). Since convergence of the TITER model took ca. 24 hours to complete, we decided to forego the training and use of 32 individual models with which a prediction is made due to computational requirements. The use of 32 independent neural networks is cited to have further improved results by Zhang et al. [2017], likely due to reduction of the variance error. Nonetheless, it is clear that this step would not close the performance gap between a single TITER model and the TIS Transformer model. All scripts used to perform the benchmark are found in the public GitHub repository `https://github.com/jdcla/TIS_transformer/tree/main/scripts/benchmarks`. By cross-referencing the total number of model weights we have verified the correct implementation of each network architecture.

2.3. Loss function

We hypothesize that weighing the loss function only has an effect for prediction tasks featuring fewer data samples, where the adjusted loss forces the learning process to focus on certain correlations more.

3. Results analysis

3.1. Rank (k) and 'false positives'

With hundreds of millions of predictions, it is necessary to select only a subset of predictions for further analysis. Following the total number of Ensembl TIS annotations k unique to each chromosome, we have determined a custom ranking for the predictions of each chromosome that is scaled to this number (rank k). For a chromosome with 1000 positively annotated TIS, the highest ranking output gets rank (k): 0, the 500th highest output rank(k): 0.5, and the 2000th highest prediction a rank (k): 2. Applying this ranking, it is possible to get a quick idea on how the model prediction compares to other predictions within the chromosome. In general, false positives refer to positive predictions with rank (k) < 1 that were not previously annotated by Ensembl.

3.2. pBLAST

To cross-reference model predictions that are not featured by Ensembl, we evaluated the CDSs resulting from predicted TISs using pBLAST. These were queried against Swiss-Prot, TrEMBL (mammals) and all supplementary isoforms. pBLAST matches were filtered following three constraints based on various properties: a match requires to be at least 95% identical to the query sequence, a maximum difference in length between the query and match of 5%, and a maximum difference in distance between the aligned start and stop sites of 5% of their total length. We are aware that given constraints are not perfect. However, constraints were tuned by evaluating the total number of matches returned when evaluating the pBLAST results on the annotations provided by Ensembl, and found to return a large portion of the correct proteins without including some obvious false positives.

3.3. Online result browser

In addition to featuring the raw results, the code and the scripts used to obtain the results on the public GitHub repository, we host an online server that provides a more accessible approach towards making our findings open to the public (Supplementary Figures A1–3). The tool is accessible through `https://jdcla.ugent.be/TIS_Transformer`. The link is furthermore linked through our public GitHub repository at `https://github.com/jdcla/TIS_transformer`. With this, we hope to attract a larger group of users that is otherwise not experienced with coding or data manipulation.

The result browser allows the user to filter predictions based on various features. Currently implemented filters are: gene/transcript name, ORF type, ORF length, Ensembl annotation, transcript type, prediction rank, and number of matches on transcript. The query returns a table of all matches, and all related information of the TISs and resulting CDSs, for easy download and visualisation. To illustrate, one can easily collect the small ORFs on non-coding sequences, all transcripts featuring multiple TISs, and transcripts featuring upstream ORFs.

4. Supplementary Tables

Table A1: **Overview of studies on TIS annotation using sequence information.** For every study is given, the year of publication, the name of the tool, the machine learning approach (SVM: Support Vector Machine, NN: Neural Network), the size of the input sequence around the candidate TIS, the total number of model parameters, the total number of data samples used in the study, and the location of public code repository. '-' denotes that the value does not apply. '?' is used when the answer is unclear from the manuscript.

Author	Year	Model name	Type	Input size	# parameters	# Samples	Code Repo
Zien et al. [2000]	2000	-	SVM	200nt	Variable	13,503	-
Saeys et al. [2007]	2007	StartScan	Varia	200nt	-	1,267,701	-
Chen et al. [2014]	2014	iTIS-PseTNC	SVM	198nt	Variable	2,318	-
Kabir et al. [2015]	2015	iTIS-PseKNC	SVM	198nt	Variable	2,318	-
Goel et al. [2020]	2020	-	SVM	303nt	Variable	3,020	-
Zhang et al. [2017]	2017	TITER	NN	203nt	∼431K	104,675	GitHub
Zuallaert et al. [2018]	2018	TISRover	NN	203nt	∼240K	94,642	-
Kalkatawi et al. [2019]	2019	DeepGSR	NN	203nt	∼181M	28,244	Zenodo
Wei et al. [2021]	2021	DeepTIS	NN	?	?	115,728	GitHub
Clauwaert et al.	2022	TIS Transf.	NN	Variable	∼118K/356K	431,011,438	GitHub

Table A2: **Model performances for the varying models used to remap the human proteome.** The test and validation sets refer the the contig identifiers. The training set uses all remaining contigs. pBLAST refers to the fraction of false positives TISs (rank (k) < 1) that return a match when performing pBLAST search on their resulting CDSs.

Model name	# parameters	Val. set	Test set	ROC AUC	PR AUC	pBLAST
TIS Transformer S	∼41K	2, 14	1, 7, 13, 19	0.9998	0.7541	-
TIS Transformer (1)	∼118K	2, 14	1, 7, 13, 19	0.9999	0.8143	-
TIS Transformer (2)	∼118K	1, 13	2, 8, 14, 20	0.9999	0.8437	-
TIS Transformer (3)	∼118K	1, 13	3, 9, 15, 21	0.9999	0.8183	-
TIS Transformer (4)	∼118K	1, 13	4, 10, 16, 22	0.9999	0.8225	-
TIS Transformer (5)	∼118K	1, 13	5, 11, 17, X	0.9999	0.8392	-
TIS Transformer (6)	∼118K	1, 13	6, 12, 18, Y	0.9999	0.8250	-
TIS Transformer L (1)	∼356K	2, 14	1, 7, 13, 19	0.9999	0.8292	0.34
TIS Transformer L (2)	∼356K	1, 13	2, 8, 14, 20	0.9999	0.8535	0.33
TIS Transformer L (3)	∼356K	1, 13	3, 9, 15, 21	0.9999	0.8401	0.34
TIS Transformer L (4)	∼356K	1, 13	4, 10, 16, 22	0.9999	0.8328	0.36
TIS Transformer L (5)	∼356K	1, 13	5, 11, 17, X	0.9999	0.8409	0.33
TIS Transformer L (6)	∼356K	1, 13	6, 12, 18, Y	0.9999	0.8355	0.38

Table A3: **Overview of the hyperparameters that define the TIS Transformer S(mall) model architecture**. This model was used as a step to compare different architectures. Also given are the keys used to define the model using the code at `https://github.com/jdcla/TIS_transformer`

Hyperparameter	Argument	Value	Hyperparameter	Argument	Value
learning rate	–lr	0.001	dim. of heads	–dim_head	
dim. of hidden state	–dim	20	kernel function	–kernel_fn	torch.nn.Relu()
layers	–depth	4	local window size	–local_window_size	256
attention heads (layer)	–heads	4	local attention heads	–local_attn_heads	3
Total trainable model parameters		∼41K			

Table A4: **Overview of the hyperparameters that define the TIS Transformer model architecture.** Also given are the keys used to define the model using the code at `https://github.com/jdcla/TIS_transformer`

Hyperparameter	argument	value	Hyperparameter	argument	value
learning rate	–lr	0.001	dim. of heads	–dim_head	16
dim. of hidden state	–dim	30	kernel function	–kernel_fn	torch.nn.Relu()
layers	–depth	6	local window size	–local_window_size	256
attention heads (layer)	–heads	6	local attention heads	–local_attn_heads	4
Total trainable model parameters		∼118K			

Table A5: **Overview of the hyperparameters that define the TIS Transformer L(arge) model architecture**. This model was used as a step to compare different architectures. Also given are the keys used to define the model using the code at `https://github.com/jdcla/TIS_transformer`

Hyperparameter	Argument	Value	Hyperparameter	Argument	Value
learning rate	–lr	0.001	dim. of heads	–dim_head	16
dim. of hidden state	–dim	48	kernel function	–kernel_fn	torch.nn.Relu()
layers	–depth	8	local window size	–local_window_size	256
attention heads (layer)	–heads	8	local attention heads	–local_attn_heads	5
Total trainable model parameters		∼356K			

Table A6: **A set of ORF annotated sequences that have been recently added to GENCODE as part of Ribo-seq studies.** The list has been retrieved from a recent publication on the advancement of ORF detection through a community-led framework Mudge et al. [2022]. uORF: upstream open reading frame; uoORF: upstream overlapping open reading frame.

Gene	Transcript	ORF biotype	CDS length	Rank (k)	
ENSG00000288654	ENST00000677770	uORF	25aa	1.61	-
ENSG00000288657	ENST00000678782	uORF	23aa	0.80	-
ENSG00000288666	ENST00000677315	uORF	59aa	1.21	-
ENSG00000288678	ENST00000679970	uORF	21aa	1.02	-
ENSG00000288677	ENST00000518377	uORF	34aa	2.75	-
ENSG00000288708	ENST00000683730	uORF	29aa	2.29	-
ENSG00000289490	ENST00000689938	uORF	18aa	2.42	-
ENSG00000289360	ENST00000685210	uORF	25aa	1.82	-
ENSG00000288914	ENST00000688005	uoORF	32aa	1.52	-
ENSG00000289025	ENST00000685402	uORF	17aa	1.28	-
ENSG00000140521	ENST00000650303	uoORF	261aa	>3	Model suggests alt. 241aa peptide
ENSG00000288528	ENST00000674075	uORF	32aa	1.83	-
ENSG00000288529	ENST00000674115	uORF	42aa	0.90	-
ENSG00000255529	ENST00000649091	uoORF	86aa	1.14	-
ENSG00000288546	ENST00000674331	uORF	40aa	-	Transcript not present in GRCh38v107
ENSG00000288645	ENST00000676296	uoORF	129aa	1.27	-
ENSG00000288623	ENST00000675818	uoORF	86aa	1.04	-
ENSG00000288614	ENST00000675098	uORF	39aa	0.99	-
ENSG00000288652	ENST00000675347	uORF	68aa	2.02	-
ENSG00000288645	ENST00000676205	uoORF	45aa	0.64	-
ENSG00000288618	ENST00000675181	uORF	32aa	1.31	-
ENSG00000288642	ENST00000617114	uoORF	103aa	1.19	-
ENSG00000288634	ENST00000674552	uORF	16aa	1.48	-
ENSG00000288633	ENST00000676334	uORF	20aa	2.00	-
ENSG00000288632	ENST00000675268	uORF	52aa	0.88	-

5. Supplementary Figures

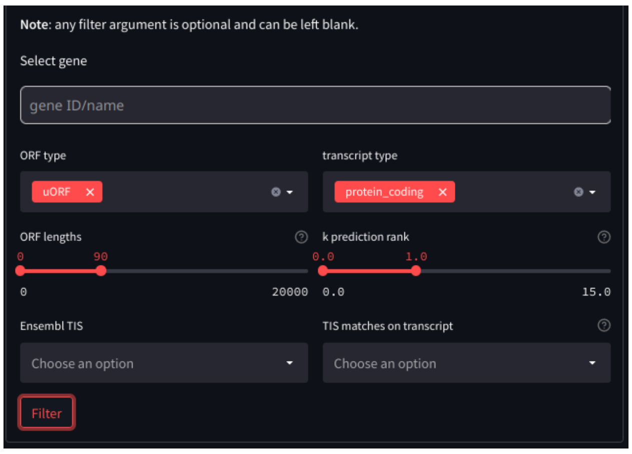

Figure A1: **A screenshot of the result browser at `https://jdcla.ugent.be/TIS_transformer` featuring multiple filter arguments.**

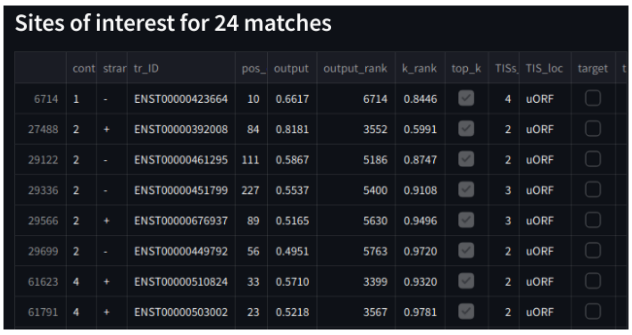

Sites of interest for 24 matches

	cont	strar	tr_ID	pos_	output	output_rank	k_rank	top_k	TISs_	TIS_loc	target	t
6714	1	-	ENST00000423664	10	0.6617	6714	0.8446	☑	4	uORF	☐	
27488	2	+	ENST00000392008	84	0.8181	3552	0.5991	☑	2	uORF	☐	
29122	2	-	ENST00000461295	111	0.5867	5186	0.8747	☑	2	uORF	☐	
29336	2	-	ENST00000451799	227	0.5537	5400	0.9108	☑	3	uORF	☐	
29566	2	+	ENST00000676937	89	0.5165	5630	0.9496	☑	3	uORF	☐	
29699	2	-	ENST00000449792	56	0.4951	5763	0.9720	☑	2	uORF	☐	
61623	4	+	ENST00000510824	33	0.5710	3399	0.9320	☑	2	uORF	☐	
61791	4	+	ENST00000503002	23	0.5218	3567	0.9781	☑	2	uORF	☐	

Figure A2: **A screenshot of part of the resulting table at `https://jdcla.ugent.be/TIS_transformer` after filtering results as featured in Figure A1**

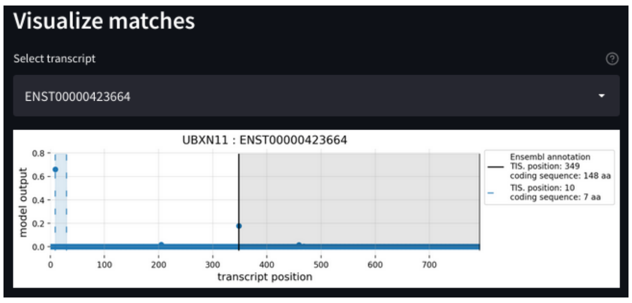

Figure A3: **A screenshot of a visualization offered at `https://jdcla.ugent.be/TIS_transformer` of one of the predicted TIS that matches the filter arguments given in Figure A1**

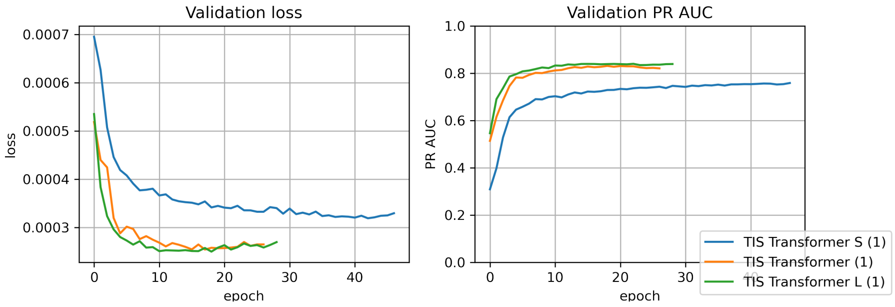

Figure A4: **The loss and PR AUC curves of three model architectures trained for annotating TISs.** The validation and test sets used are chromosomes 2, 14 and chromosomes 1, 7, 13, 19, respectively. The hyperparameters for each model are given in Table A3, A4, A5

.

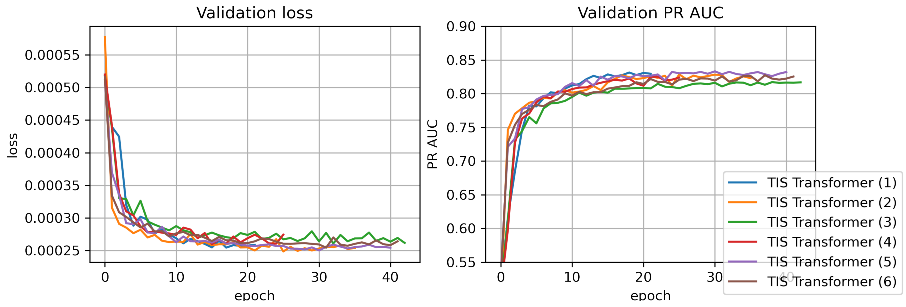

Figure A5: **The loss and PR AUC curves for the models trained on the TIS annotation task.** Each model has a different set of chromosomes for the train/test/validation set, as given in Table A2.

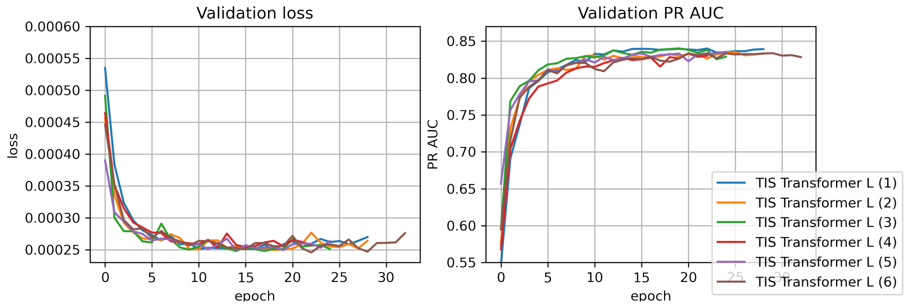

Figure A6: **The loss and PR AUC curves for the models trained on the TIS annotation task.** Each model has a different set of chromosomes for the train/test/validation set, as given in Table A2.

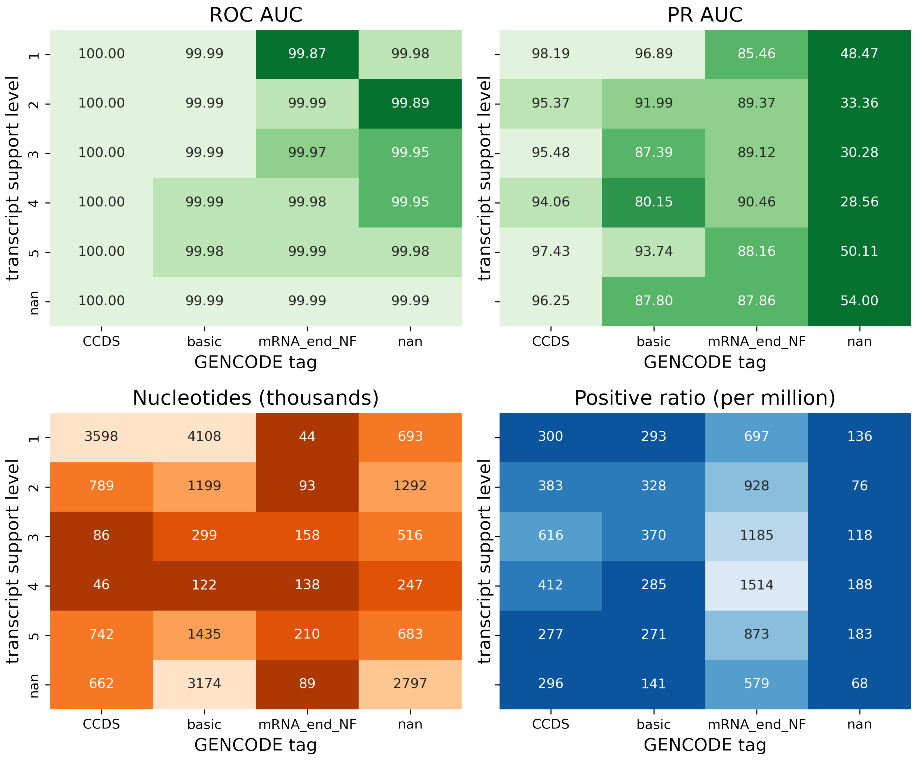

Figure A7: **Model performances and input information for the inputs binned by transcript support level and tags given to the annotated translation initiation sites.** ROC AUC and PR AUC performances (top) are given as well as the total number of annotated TISs (by Ensembl) and ratio of positive samples (w.r.t. negative samples). Values are obtained by binning predictions per transcripts according to transcript support level and by binning the predictions by tags given to the annotated translation initiation site.

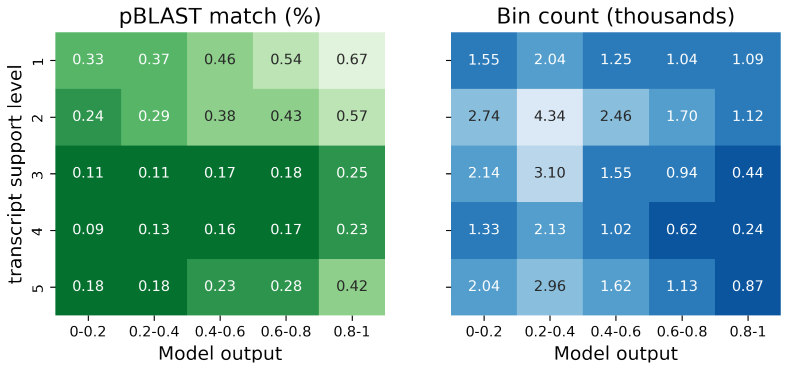

Figure A8: **Fraction of good pBLAST matches for novel model predictions binned by transcript support level and model output range.** Evaluated predictions have been limited to those within rank (k) < 1.5 (to equalize the leftmost bin size) and exclude those previously annotated by Ensembl. (**left**) The fraction of TISs that result in a coding sequence with a strong pBLAST match. (**right**) The number of samples in each bin.

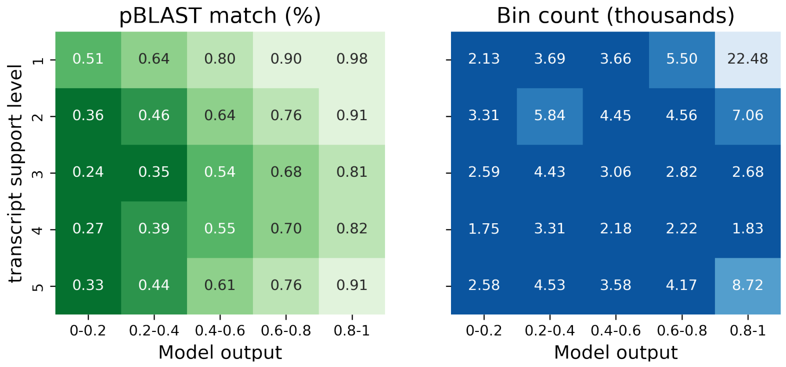

Figure A9: **Fraction of good pBLAST matches for model predictions binned by transcript support level and model output range.** Evaluated predictions have been limited to those within rank (k) < 1.5 (to equalize the leftmost bin size) and include those previously annotated by Ensembl. (**left**) The fraction of TISs that result in a coding sequence with a strong pBLAST match. (**right**) The number of samples in each bin.

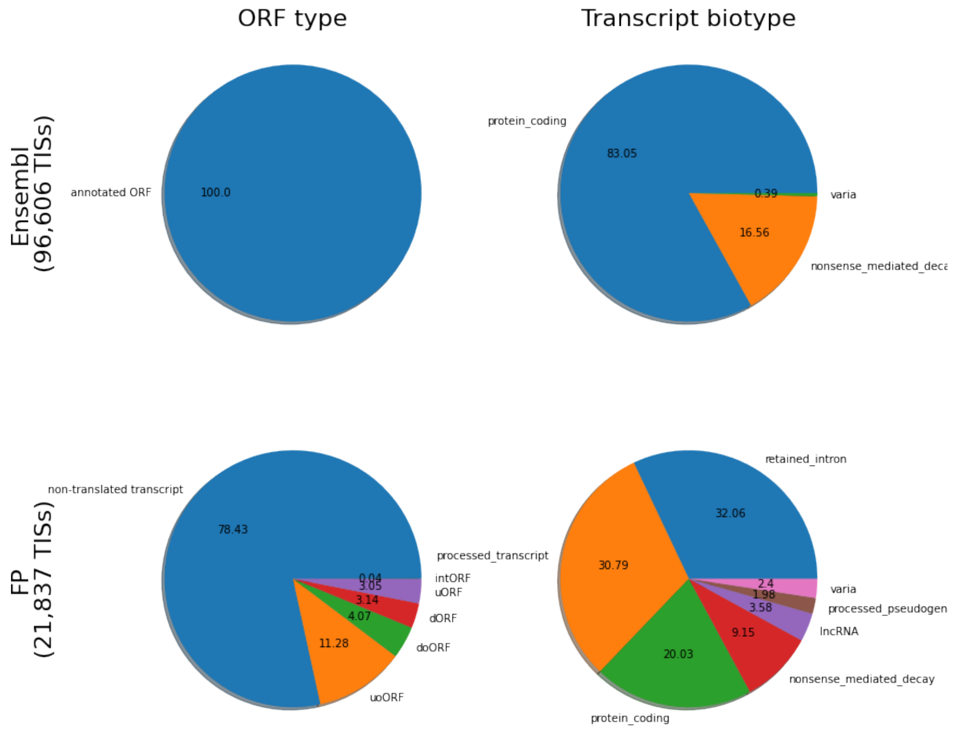

Figure A10: **Property distributions between annotated and newly predicted TISs.** These predictions constitute those that are not annotated by Ensembl and have a rank (k) < 1, referred to as the false positive (FP) set. For both groups the ORF type of the annotation and the biotype of the transcript are given. FP annotations on protein coding transcripts are either additional CDSs detected alongside the canonical annotation, or alternative TIS.

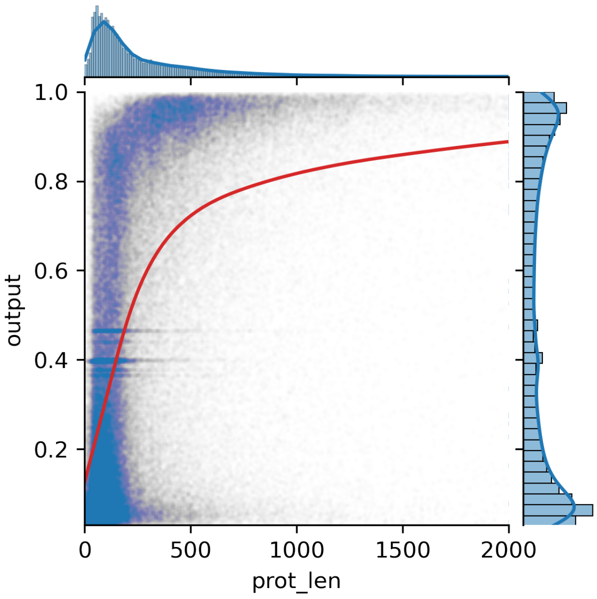

Figure A11: **Correlation between the model output for a given translation initiation site and the length of its resulting protein.** The trend line (red line) is obtained using LOWESS.

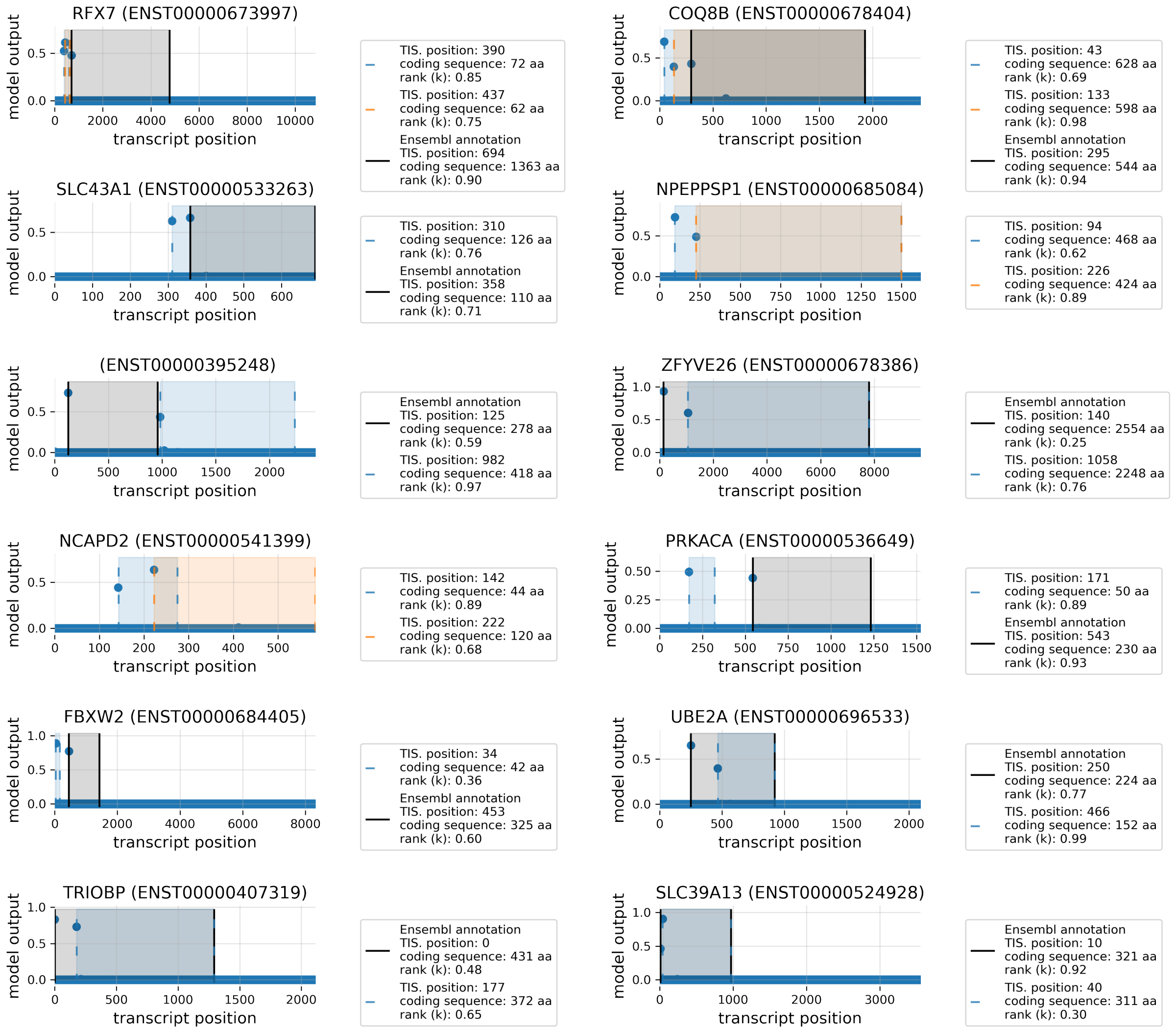

Figure A12: **Model predictions on several transcripts with multiple high-ranking TIS positions.** Shown are the model outputs (y-axis) for each position of the transcript (x-axis). For high TIS predictions, the bounds of the resulting CDS are given, as well as their length and prediction rank (n-highest prediction) on the chromosome. When a CDS is present in Ensembl, the bounds are represented by full black lines.

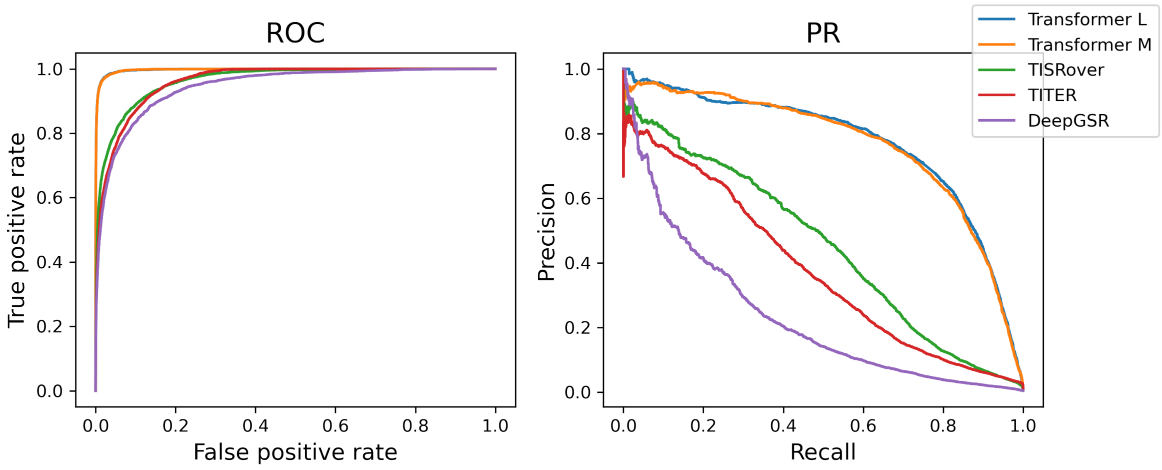

Figure A13: **Receiver operating characteristics (ROC) and precision-recall (PR) curves of the models trained for the benchmark.**

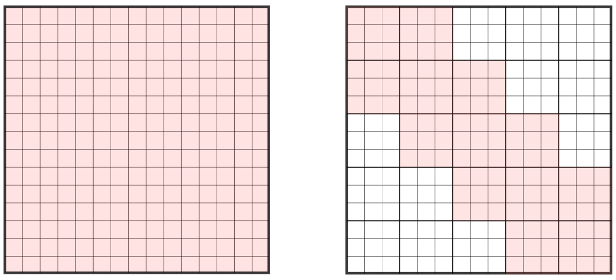

Figure A14: **Attention schemes used by the attention heads in the model. (left)** full attention heads allow every input nucleotide position to attend to all other positions on the transcript. These attention heads utilize the FAVOR+ approximation algorithm to allow input sequences of up to 30,000 tokens. **(right)**: Local attention is implemented by dividing the attention matrix in smaller blocks on which full attention is calculated. Three blocks around the evaluated input are calculated. The window size of each block listed as 'local_window_size' in Supplementary Table A4-A6.

References

W. Chen, P.-M. Feng, E.-Z. Deng, H. Lin, and K.-C. Chou. iTIS-PseTNC: A sequence-based predictor for identifying translation initiation site in human genes using pseudo trinucleotide composition. *Analytical Biochemistry*, 462:76–83, Oct. 2014. ISSN 0003-2697. doi: 10.1016/j.ab.2014.06.022.

K. Choromanski, V. Likhosherstov, D. Dohan, X. Song, A. Gane, T. Sarlos, P. Hawkins, J. Davis, A. Mohiuddin, L. Kaiser, D. Belanger, L. Colwell, and A. Weller. Rethinking Attention with Performers. *arXiv:2009.14794 [cs, stat]*, Mar. 2021.

N. Goel, S. Singh, and T. C. Aseri. Global sequence features based translation initiation site prediction in human genomic sequences. *Heliyon*, 6(9):e04825, Sept. 2020. ISSN 2405-8440. doi: 10.1016/j.heliyon.2020.e04825.

M. Kabir, M. Iqbal, S. Ahmad, and M. Hayat. iTIS-PseKNC: Identification of Translation Initiation Site in human genes using pseudo k-tuple nucleotides composition. *Computers in Biology and Medicine*, 66:252–257, Nov. 2015. ISSN 0010-4825. doi: 10.1016/j.compbiomed.2015.09.010.

M. Kalkatawi, A. Magana-Mora, B. Jankovic, and V. B. Bajic. DeepGSR: An optimized deep-learning structure for the recognition of genomic signals and regions. *Bioinformatics*, 35(7):1125–1132, Apr. 2019. ISSN 1367-4803. doi: 10.1093/bioinformatics/bty752.

J. M. Mudge, J. Ruiz-Orera, J. R. Prensner, M. A. Brunet, F. Calvet, I. Jungreis, J. M. Gonzalez, M. Magrane, T. F. Martinez, J. F. Schulz, Y. T. Yang, M. M. Albà, J. L. Aspden, P. V. Baranov, A. A. Bazzini, E. Bruford, M. J. Martin, L. Calviello, A.-R. Carvunis, J. Chen, J. P. Couso, E. W. Deutsch, P. Flicek, A. Frankish, M. Gerstein, N. Hubner, N. T. Ingolia, M. Kellis, G. Menschaert, R. L. Moritz, U. Ohler, X. Roucou, A. Saghatelian, J. S. Weissman, and S. van Heesch. Standardized annotation of translated open reading frames. *Nature Biotechnology*, 40(7):994–999, July 2022. ISSN 1546-1696. doi: 10.1038/s41587-022-01369-0.

Y. Saeys, T. Abeel, S. Degroeve, and Y. Van de Peer. Translation initiation site prediction on a genomic scale: Beauty in simplicity. *Bioinformatics*, 23(13):i418–i423, July 2007. ISSN 1367-4803. doi: 10.1093/bioinformatics/btm177.

J. Su, Y. Lu, S. Pan, A. Murtadha, B. Wen, and Y. Liu. RoFormer: Enhanced Transformer with Rotary Position Embedding, Aug. 2022.

C. Wei, J. Zhang, and Y. Xiguo. DeepTIS: Improved translation initiation site prediction in genomic sequence via a two-stage deep learning model. *Digital Signal Processing*, 117:103202, Oct. 2021. ISSN 1051-2004. doi: 10.1016/j.dsp.2021.103202.

S. Zhang, H. Hu, T. Jiang, L. Zhang, and J. Zeng. TITER: Predicting translation initiation sites by deep learning. *Bioinformatics*, 33(14):i234–i242, July 2017. ISSN 1367-4803. doi: 10.1093/bioinformatics/btx247.

A. Zien, G. Rätsch, S. Mika, B. Schölkopf, T. Lengauer, and K.-R. Müller. Engineering support vector machine kernels that recognize translation initiation sites. *Bioinformatics*, 16(9):799–807, Sept. 2000. ISSN 1367-4803. doi: 10.1093/bioinformatics/16.9.799.

J. Zuallaert, M. Kim, A. Soete, Y. Saeys, and W. D. Neve. TISRover: ConvNets learn biologically relevant features for effective translation initiation site prediction. *International Journal of Data Mining and Bioinformatics*, 20(3):267–284, Jan. 2018. ISSN 1748-5673. doi: 10.1504/IJDMB.2018.094781.
